# Frameshift and wild-type proteins are often highly similar because the genetic code and genomes were optimized for frameshift tolerance

**DOI:** 10.1186/s12864-022-08435-6

**Published:** 2022-06-02

**Authors:** Xiaolong Wang, Quanjiang Dong, Gang Chen, Jianye Zhang, Yongqiang Liu, Yujia Cai

**Affiliations:** 1 Department of Biotechnology, College of Marine Life Sciences, Ocean University of China, No. 5 Yushan Road, Shandong Qingdao, 266003 P. R. China; 2 Qingdao Municipal Hospital, Qingdao, Shandong 266003 P. R. China

## Abstract

Frameshift mutations have been considered of significant importance for the molecular evolution of proteins and their coding genes, while frameshift protein sequences encoded in the alternative reading frames of coding genes have been considered to be meaningless. However, functional frameshifts have been found widely existing. It was puzzling how a frameshift protein kept its structure and functionality while substantial changes occurred in its primary amino-acid sequence. This study shows that the similarities among frameshifts and wild types are higher than random similarities and are determined at different levels. Frameshift substitutions are more conservative than random substitutions in the standard genetic code (SGC). The frameshift substitutions score of SGC ranks in the top 2.0-3.5% of alternative genetic codes, showing that SGC is nearly optimal for frameshift tolerance. In many genes and certain genomes, frameshift-resistant codons and codon pairs appear more frequently than expected, suggesting that frameshift tolerance is achieved through not only the optimality of the genetic code but, more importantly, the further optimization of a specific gene or genome through the usages of codons/codon pairs, which sheds light on the role of frameshift mutations in molecular and genomic evolution.

**Supplementary Information:**

The online version contains supplementary material available at 10.1186/s12864-022-08435-6.

## Background

The genetic code was deciphered in the early 1960s [1]. The standard genetic code (SGC) consists of 64 triplet codons, 61 sense codons for the twenty amino acids (AAs), and three nonsense codons for stop signals. Dozens of alternative genetic codes have been reported in all three phylogenetic domains of life [2]. As shown in the list of genetic codes (https://www.ncbi.nlm.nih.gov/Taxonomy/Utils/wprintgc.cgi) maintained by the National Center for Biotechnology Information (NCBI), all the alternative genetic codes have only one to six differences from the standard code. All these natural genetic codes share several important properties: (1) the genetic code is universal, with only a few variations found in some organelles or organisms, such as mitochondrion, archaea, yeast, and ciliates [3]; (2) the triplet codons are redundant, degenerate, and changes at the third base of codons, known as the interchangeable position, are generally synonymous; (3) in a coding DNA sequence (CDS), an insertion or deletion (InDel) causes a frameshift mutation if its size is not a multiple of three.

It has been reported that the natural genetic code was optimized for translational error minimization, which is being extremely efficient at minimizing the effect of point mutation or mistranslation errors and is optimal for kinetic energy conservation in polypeptide chains [4–7]. Moreover, it was discovered that the SGC resists frameshift errors by increasing the probability that a stop signal is encountered upon frameshifting because frameshifted codons for abundant amino acids overlap with stop codons [8].

A frameshift mutation alters the reading frame of a coding gene and may produce frameshift proteins (frameshifts). Frameshifts have been considered mostly meaningless since they look completely different from the wild type and are often interrupted by many stop signals. A frameshifted gene yields truncated, non-functional, and potentially cytotoxic peptides [9]. Until the end of the last century, frameshift mutations were generally considered harmful and of little importance to the evolution of protein-coding genes. In the first two decades of this century, however, it was widely found that frameshifted genes can sometimes be expressed through several special mechanisms, such as translational readthrough [10–12], ribosomal frameshifting [13–15], reading frame transition [14], and genetic recoding [16]. Moreover, frameshifted coding genes can be retained for millions of years and enable the acquisition of new functions [17].

Moreover, there have been a handful of cases of functional frameshifts that retain their function. For example, Hahn and Lee identified nine frameshift homologs between humans and chimpanzees by collecting human coding exons bearing InDels compared with the chimpanzee genome, some of which seem to be functional in both species [18]. By blastp searching the protein database using specialized scoring matrices designed for frameshifts, Claverie identified several functional frameshifts in bacteria, yeast, humans, and rats [19]. Recently, Huang et al. [20] showed that frameshift proteins of a bacteria toxin retain the same function. Moreover, it has also been widely discovered that frameshift mutations may lead to functional divergence [17], novel genes [21], or overlapping genes in viruses [22], bacteria [23], and even humans [24].

As is well known, a protein can become dysfunctional by changing even one residue, so it is puzzling how a frameshift protein can maintain the integrity of its tertiary structure and function while substantial changes occur in its primary sequence. Based on ClustalW alignments, we have observed high similarities among frameshifts and wild-type protein sequences [25]; recently, Bartonek et al. further proved that frameshifting preserves key physicochemical properties of proteins [26]. Inspired by their work and peer reviewers’ comments, we realized that our previous similarity calculations were overestimated due to the gappy alignments. ClustalW [27] works well in common protein sequences but is not designed for aligning frameshift protein sequences. Actually, there is no existing method suitable for aligning frameshift protein sequences. Therefore, we developed a specialized alignment method for frameshift protein sequences (FrameAlign). Using FrameAlign, we reanalyzed the data and proved that frameshift and wild-type protein sequences are often highly similar. Furthermore, we proved that the SGC is nearly optimal for frameshift tolerance, and certain genes and genomes were further optimized to enhance their tolerance to frameshift mutations through biased usage of codons/codon pairs, which shed light on the role of frameshift mutations in molecular and genomic evolution.

## Materials and methods

### Protein-coding DNA sequences

All reference coding sequences (CDSs) in ten model species, including *Escherichia coli, Saccharomyces cerevisiae, Arabidopsis thaliana, Caenorhabditis elegans, Drosophila melanogaster, Danio rerio, Xenopus tropicalis, Mus musculus*, *Pan troglodytes*, and *Homo sapiens*, were retrieved from GenBank Genome Database. Program RandomCDSs.java produced ten thousand sets of CDSs, each containing three CDSs and each CDS containing 300 or 500 random sense codons.

### Aligning and computing the similarities of wild types and frameshifts

For a given CDS, let *δ*_*ij*_ be the pairwise similarities of its three translations, *i, j =* 1, 2, 3, *i ≠ j*, *δ*_*ij*_ = *δ*_*ji*_. The average similarity among the frameshifts and the wild type is defined as the shiftability of protein-coding genes (*δ*)*,*

$$\delta =\frac{1}{3}\left({\delta}_{12}+{\delta}_{13}+{\delta}_{23}\right)$$

Shiftability is a quantitative measurement of frameshift tolerability. As frameshifting occurs between any two of the three reading frames, *δ*_12_, *δ*_13_, and *δ*_23_ are all considered in the formula.

Program Similarity.java batch translates CDSs and computes the pairwise similarities among the three translations, in which CDSs are translated using the SGC in the different reading frames of the sense strand, and the three different translations are aligned by different methods, including ClustalW2, MSA, and FrameAlign. To calculate pairwise similarity, a pair of matched AAs in a pairwise alignment is considered conserved if their substitution score is ≥0 in the scoring matrix GON250, i.e., gaps and negative scores are considered different. The percent of conserved sites gives the pairwise similarity between a pair of protein sequences.

Similarity.java has an option to translate internal stop codon into AAs using a set of readthrough rules (Table 1). Translational readthrough occurs upon the suppressor tRNA activity with an anticodon matching a stop codon [12]. Many studies have shown that translational readthrough occurs in prokaryotes and eukaryotes, from *E. coli* to humans, while the readthrough rules may vary among different species [28]. In *E. coli*, nonsense suppression tRNAs reported includes amber suppressors (*supD* [29], *supE* [30], *supF* [31]), ochre suppressors (*supG* [32]), and opal suppressors (*supU* [31], *su9* [33]). The suppressor tRNAs were summarized as a list of readthrough rules. If the user selects the option “readthrough”, these rules are adopted to read through the stop codons.

**Table 1:** The readthrough rules derived from natural suppressor tRNAs for nonsense mutations

Site	tRNA (AA)	Codon
*supD*	Ser (S)	UAG
*supE*	Gln (Q)	UAG
*supF*	Tyr (Y)	UAG
*supG*	Lys (K)	UAA
*supU*	Trp (W)	UGA

### FrameAlign: aligning of frameshifts and wild-type protein sequences

A wild-type protein-coding sequence consisting of *n* triplet codons is written as,

$${B}_1{B}_2{B}_3\mid {\boldsymbol{B}}_{\boldsymbol{4}}{\boldsymbol{B}}_{\boldsymbol{5}}{\boldsymbol{B}}_{\boldsymbol{6}} \mid {B}_7{B}_8{B}_9\mid \dots \mid {B}_{3i\hbox{-} 2}{B}_{3i\hbox{-} 1}{B}_{3i}\left|{\boldsymbol{B}}_{\boldsymbol{3i}+\boldsymbol{1}}{\boldsymbol{B}}_{\boldsymbol{3i}+\boldsymbol{2}}{\boldsymbol{B}}_{\boldsymbol{3i}+\boldsymbol{3}}\right|\dots \mid {B}_{3n\hbox{-} 2}{B}_{3n\hbox{-} 1}{B}_{3n}$$

where *B*_*k*_ ∈{*A, G, U, C*}; *i = 1… n*; *k =* 1…3*n*. Each pair of neighboring codons are separated by a bar to show the native reading frame. Its encoded wild-type protein sequence (*WT*), consisting of *n* amino acids, can be written as,

$$\boldsymbol{WT}:{A}_{B_1{B}_2{B}_3}\ {A}_{B_4{B}_5{B}_6}...{A}_{B_{3i-2}{B}_{3i-1}{B}_{3i}}\ {A}_{B_{3i+ 1}{B}_{3i+ 2}{B}_{3i+ 3}}... {A}_{B_{3n-5}{B}_{3n-4}{B}_{3n-3}}\ {A}_{B_{3n-2}{B}_{3n-1}{B}_{3n}}$$

where $A_{B_{3i-2} B_{3i-1} B_{3i}}$ ∈{*A, C, D, E, F, G, H, I, K, L, M, N, P, Q, R, S, T, V, W, Y*}, represents the amino acid encoded by the *i*^th^ codon (*B*_*3i-2*_*B*_*3i-1*_*B*_*3i*_). If a frameshift is caused by deleting or inserting one or two bases in the start codon, there are only four cases:

1. Delete one (− 1)*: B*_*2*_*B*_*3*_***B***_***4***_***| B***_***5***_***B***_***6***_*B*_*7*_*|* … *| B*_*3i-1*_*B*_*3i*_***B***_***3i + 1 |***_***B***_***3i + 2***_***B***_***3i + 3***_*B*_*3i + 4*_*| …*
2. Delete two (− 2)*: B*_*3*_***B***_***4***_***B***_***5***_***| B***_***6***_*B*_*7*_*B*_*8*_*|* … *| B*_*3i*_***B***_***3i + 1***_***B***_***3i + 2 |***_***B***_***3i + 3***_*B*_*3i + 4*_*B*_*3i + 5*_*| …*
3. Insert one (+ 1)*: B*_*0*_*B*_*1*_*B*_*2*_*| B*_*3*_***B***_***4***_***B***_***5***_***| B***_***6***_*B*_*7*_*B*_*8*_*|* … *| B*_*3i-3*_*B*_*3i-2*_*B*_*3i-1*_*| B*_*3i*_***B***_***3i + 1***_***B***_***3i + 2***_*| …*
4. Insert two (+ 2)*: B*_*− 1*_*B*_*0*_*B*_*1*_*| B*_*2*_*B*_*3*_***B***_***4***_***| B***_***5***_***B***_***6***_*B*_*7*_*|* … *| B*_*3i-4*_*B*_*3i-3*_***B***_***3i-2***_***| B***_***3i-1***_***B***_***3i***_*B*_*3i + 1*_*| …*

If a frameshift mutation occurs at any location between the first and the *i*^th^ codon, the (*i* + 1)^*th*^ codon (***B***_***3i + 1***_***B***_***3i + 2***_***B***_***3i + 3***_) has only two possible changes:

1. Forward frameshifting (*FF*): $A_{B_{3i + 2} B_{3i + 3} B_{3i + 4}}$
2. Reverse frameshifting (*RF*): $A_{B_{3i} B_{3i + 1} B_{3i + 2}}$ 

This continues for each codon downstream, resulting in two frameshifts, denoted as *FF* and *RF*,

$${\displaystyle \begin{array}{l}\boldsymbol{FF}:{A}_{B_2{B}_3{B}_4}\ {A}_{B_5{B}_6{B}_7}\dots {A}_{B_{3i\hbox{-} 1}{B}_{3i}{B}_{3i+ 1}}\ {A}_{B_{3i+ 2}{B}_{3i+ 3}{B}_{3i+ 4}}\dots {A}_{B_{3n\hbox{-} 7}{B}_{3n\hbox{-} 6}{B}_{3n\hbox{-} 5}}\ {A}_{B_{3n\hbox{-} 4}{B}_{3n\hbox{-} 3}{B}_{3n\hbox{-} 2}}\kern0.5em \left[{}_{B_{3n\hbox{-} 1}{B}_{3n}}\right]\\ {}\boldsymbol{RF}:{A}_{B_3{B}_4{B}_5}\ {A}_{B_6{B}_7{B}_8}\dots {A}_{B_{3i\hbox{-} 3}{B}_{3i\hbox{-} 2}{B}_{3i\hbox{-} 1}}{A}_{B_{3i}{B}_{3i+ 1}{B}_{3i+ 2}}\dots {A}_{B_{3n\hbox{-} 6}{B}_{3n\hbox{-} 5}{B}_{3n\hbox{-} 4}}{A}_{B_{3n\hbox{-} 3}{B}_{3n\hbox{-} 2}{B}_{3n\hbox{-} 1}}\left[{}_{B_{3n}}\right]\end{array}}$$

The last codon of *FF* or *RF* shown in square brackets is incomplete and was deleted in the computation process. The *i*^th^ codon of the frameshifts (*B*_*3i + 2*_*B*_*3i + 3*_*B*_*3i + 4*_ for *FF* or *B*_*3i*_*B*_*3i + 1*_*B*_*3i + 2*_ for *RF*) has two bases overlapping with the (*i* + 1)^*th*^ codon of *WT* (*B*_*3i + 1*_*B*_*3i + 2*_*B*_*3i + 3*_); the encoded amino acids ($A_{B_{3i + 2} B_{3i + 3} B_{3i + 4}}$, $A_{B_{3i} B_{3i + 1} B_{3i + 2}}$, and $A_{B_{3i + 1} B_{3i + 2} B_{3i + 3}}$) are likely similar to each other because similar codons encode amino acids with related physicochemical properties [4]. Except for the interchangeable codons, amino acids with similar physicochemical properties are located in close proximity to each other in the codon table, and the coding codons usually differ by only one base substitution, e.g., hydrophobic amino acids are usually coded by codons with thymine (T) in the second position and hydrophilic amino acids by those with adenine (A) in this position [4].

Moreover, we noticed that shifted codons also often encode similar amino acids, e.g., aac (N) and act (T) are both small amino acids, while gtt (V) and ttg (L) are both aliphatic (with the shared bases underlined), and the corresponding amino acid substitution scores are positive (see The genetic code was optimized for frameshift tolerance section for the detailed analyses of shifted codons). Compared with the wild-type CDS, the frameshifted CDS consists of shifted codons successively throughout the whole sequence, so the encoded amino acid sequence (i.e., the frameshift translation) is also likely to be similar to the wild-type translation.

However, as shown in the following schematic expressions, *WT*, *FF*, and *RF* can only be aligned correctly in three pairwise alignments, but not in a multiple sequence alignment. Therefore, common aligners are not suitable for aligning frameshifts. This frameshift alignment method and these pairwise alignments are referred to as FrameAlign.

1. ***WT*vs.*FF:*** insert one gap at the end of *FF*.

(2). ***WT*vs.*RF:*** insert one gap at the beginning of *RF*.

(3). ***FF*vs.*RF*:** no gaps are needed.

$${\displaystyle \begin{array}{l}\boldsymbol{FF}:\kern0.5em {A}_{B_2{B}_3{B}_4}\ {A}_{B_5{B}_6{B}_7}\dots {A}_{B_{3i-1}{B}_{3i}\kern0.5em {B}_{3i+1}}\ {A}_{B_{3i+2}{B}_{3i+3}{B}_{3i+4}}\dots {A}_{B_{3n-7}{B}_{3n-6}{B}_{3n-5}}\kern0.5em {A}_{B_{3n-4}{B}_{3n-3}{B}_{3n-2}}\\ {}\boldsymbol{RF}:\kern0.5em {A}_{B_3{B}_4{B}_5}\ {A}_{B_6{B}_7{B}_8}\dots {A}_{B_{3i}{B}_{3i+1}{B}_{3i+2}}\ {A}_{B_{3i+3}{B}_{3i+4}{B}_{3i+5}}\dots {A}_{B_{3n-6}{B}_{3n-5}{B}_{3n-4}}\ {A}_{B_{3n-3}{B}_{3n-2}{B}_{3n-1}}\end{array}}$$

### Computational analysis of frameshift codon substitutions

According to whether the encoded AA is changed or not, codon substitutions have been classified into synonymous substitutions (SSs) and nonsynonymous substitutions (NSSs)*.* Based on the above analysis in FrameAlign: aligning of frameshifts and wild-type protein sequences section, we further classified codon substitutions into three subtypes:

1. Random substitutions (RCSs) are produced by randomly changing all three bases of the codons; there are 64 × 64 = 4096 possible RCSs.
2. Interchangeable substitutions (ICSs) are produced by randomly changing only the third position of the codons; there are 64 × 4 = 256 possible ICSs.
3. Frameshift substitutions (FCSs) are produced by forward or reverse shifting. Each codon has four forward and four reverse FCSs, and there are 64 × 8 = 512 possible FCSs.

In most cases, all three bases in the frameshifted codon are changed compared with the original codon, except for triplet monomers (such as aaa, ggg). The AA substitution scores of FCSs and RCSs are defined as frameshift substitution scores (FSSs) and random substitution scores (RSSs), respectively. The sum FSS of all possible FCSs is considered a measure of the frameshift tolerability of the genetic code. Program ShiftCodons.java computes the substitution scores for each type of codon substitutions using a scoring matrix, BLOSSUM62 [34], PAM250 [35, 36], or GON250 [37].

### Computational analysis of alternative codon tables

RandomCodes.java generates random codon tables by swapping AAs assigned to the sense codons and keeping all degenerative codons synonymous (Freeland and Hurst [7]). One million random codon tables were sampled from all possible (20! = 2.43290201 × 10^18^) genetic codes randomly using a random-number-based sampling algorithm, in which the probability of an AA being swapped is proportional to its proportion in the code table. The sampling was repeated 100 times independently. AlternativeCodes.java gives all (13824) possible compatible codon tables by permuting the nucleotide in each codon position independently (Itzkovitz and Alon [8]). Each compatible code has the same number of codons per amino acid and the same impact of misread errors as the SGC. The sum FSSs for each random or compatible genetic code was computed and compared to the SGC.

### Analysis of codon pairs and their frameshift substitution scores

FrameshiftCodonPair.java computes the FSSs for all possible codon pairs. For a given codon pair, *B*_*1*_*B*_*2*_*B*_*3*_*|****B***_***4***_***B***_***5***_***B***_***6***_, its encoded AA pair is $A_{B_{\emph{1}}B_{\emph{2}}B_{\emph{3}}}A_{B_{\emph{4}}B_{\emph{5}}B_{\emph{6}}}$. There are 400 different AA pairs, 64 × 64 = 4096 different codon pairs. Similarly, the codon pair and its encoded AAs have only two types of changes in frameshifting:

1. Forward frameshifting: $$A_{B_0 B_1 B_2} A_{B_3 B_4 B_5}$$
2. Reverse frameshifting: $$A_{B_2 B_3 B_4} A_{B_5 B_6 B_7}$$

where *B*_*0*_ and *B*_*7*_ each have four choices. There are 4096 × 8 = 32,768 different codon pair frameshift substitutions (CPFSs). For each CPFSs, $A_{B_1 B_2 B_3} A_{B_4 B_5 B_6}$ was compared with shifted codon pairs to obtain their FSSs.

### Computational analysis of the usage of codon and codon pairs

The number of occurrences was counted for each codon/codon pair for each genome. The observed and expected frequencies were calculated using the Gutman and Hatfield method for each codon or codon pair, resulting in a list of 64 codons and 4096 codon pairs, each with an expected (*E*) and observed (*O*) number of occurrences, frequency, together with a value for the *χ*^2^ statistics. A codon or codon pair was identified as over-represented if *O* > *E* (or under-represented if *O* < *E*), and the average FSSs were calculated for each genome weighted by their codon or codon pair usages.

## Results and analysis

### Wild-type and frameshift translations are often highly similar

**Fig. 1:**
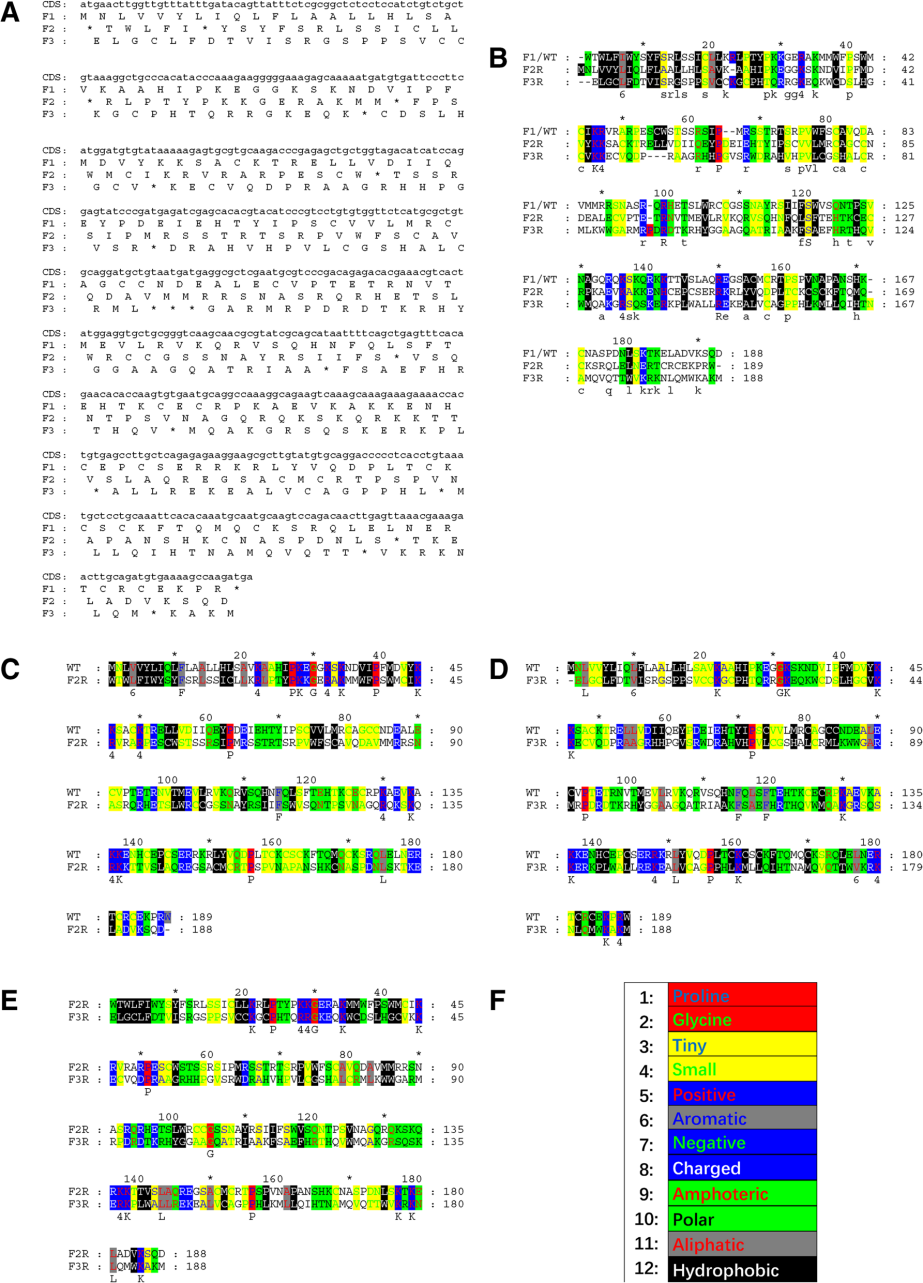
Different alignments of the three translations of zebrafish *vegfaa*. **A** The wild-type and frameshift translations of zebrafish *vegfaa*; **B** The ClustalW alignment of the three translations; **C** FrameAlign of the first and the second translations; **D** FrameAlign of the first and the third translations; **E** FrameAlign of the second and the third translations. **F** The color scheme of GeneDoc, which is used in (**B**-**E**) to color the amino acids by their physicochemical properties. CDS: coding sequence; F1: the first translation (wild type); F2: the second translation (+ 1 frameshift); F3: the third translation (+ 2 frameshift); F2R: F2 readthrough; F3R: F3 readthrough

Usually, a frameshift refers to an organism that has a frameshift mutation, i.e., having a protein-coding gene with an altered reading frame compared to the wild type; sometimes, it also refers to a putative protein sequence artificially translated from an alternative reading frame of a CDS, e.g., the second and third translations of zebrafish *vegfaa* (Fig. 1A). As described in FrameAlign: aligning of frameshifts and wild-type protein sequences section, when the CDSs are translated in the three different reading frames of the sense strand, each of them produces a set of three frame translations (*WT*, *FF,* and *RF*). In order to distinguish the two different implications of the term frameshift, hereafter, we refer to the two frameshifted protein sequences (*FF* and *RF*) as frameshift translations*.* Frameshift translations usually do not exist in nature, and they have been considered mostly meaningless since they look like random sequences.

In this section, three groups of CDSs were translated, the translations were aligned, and their similarities were calculated:

1. All available reference CDSs (real CDSs) for ten model species were translated, each producing a set of real frame translations (*WT*, *FF,* and *RF*). Each set of them was aligned using ClustalW and FrameAlign, and their similarities were calculated as real frame similarities.
2. Ten thousand random CDSs were translated, each producing a set of random frame translations (*WT*, *FF,* and *RF*). Each set of them was aligned using ClustalW, MSA, and FrameAlign, and their similarities were calculated as random frame similarities.
3. Ten thousand sets of CDSs (each containing three random CDSs) were translated in the native frame of the sense strand, each producing a set of random native translations; each set of them was aligned using ClustalW, MSA, and FrameAlign, and their similarities were calculated as random similarities.

When the frame translations were aligned using ClustalW, the estimated (hereafter est) means of real and random frame similarities are respectively 0.456 ± 0.033 and 0.452 ± 0.013 (Table 2 and S1a). But, on average, ClustalW placed 49.57 and 80.11 gaps in the real and random frame translations, respectively. Besides, the est. mean random similarity is comparable to the est. mean random frame similarity, but on average, 137.05 gaps are placed in the random native translations, indicating that these similarity calculations might be overestimated due to the alignment artifacts caused by inserting excessive gaps.

**Table 2:** The similarities of proteins and their frameshifts (aligned by ClustalW or MSA)

Type	Species	Number of CDSs	Average Similarity						Num of Gaps
			δ_**12**_	δ_**13**_	δ_**23**_	δ	MAX	MIN	
Real CDSs (ClustalW)	*H. sapiens*	71,853	0.474 ± 0.039	0.454 ± 0.046	0.433 ± 0.043	**0.464 ± 0.033**	0.890	0.271	53.3
	*P. troglodytes*	15,781	0.473 ± 0.04	0.452 ± 0.047	0.431 ± 0.042	**0.463 ± 0.034**	0.657	0.309	48.9
	*M. musculus*	27,208	0.469 ± 0.038	0.448 ± 0.046	0.43 ± 0.041	**0.459 ± 0.033**	0.739	0.286	52.5
	*X. tropicalis*	7706	0.477 ± 0.038	0.455 ± 0.044	0.439 ± 0.042	**0.466 ± 0.032**	0.638	0.320	36.8
	*D. rerio*	14,151	0.465 ± 0.036	0.443 ± 0.043	0.433 ± 0.038	**0.454 ± 0.032**	0.658	0.332	51.4
	*D. melanogaster*	23,936	0.455 ± 0.039	0.432 ± 0.045	0.426 ± 0.039	**0.444 ± 0.033**	0.702	0.250	69.4
	*C. elegans*	29,227	0.475 ± 0.037	0.444 ± 0.042	0.441 ± 0.042	**0.459 ± 0.032**	0.750	0.261	50.4
	*A. thaliana*	35,378	0.468 ± 0.038	0.439 ± 0.042	0.436 ± 0.043	**0.453 ± 0.032**	0.828	0.217	47.6
	*S. cerevisiae*	5889	0.482 ± 0.043	0.451 ± 0.042	0.463 ± 0.047	**0.467 ± 0.035**	0.692	0.259	39.7
	*E.coli*	4140	0.441 ± 0.039	0.415 ± 0.043	0.408 ± 0.042	**0.428 ± 0.032**	0.614	0.280	45.6
	Average	235,269	0.468 ± 0.039	0.443 ± 0.044	0.434 ± 0.042	**0.456 ± 0.033**	0.890^a^	0.217^a^	49.6
Random CDSs (ClustalW)	Three frames	100000 × 3	0.475 ± 0.019	0.428 ± 0.020	0.427 ± 0.020	**0.452 ± 0.013**	0.512	0.391	80.1
	Three random CDSs	100000 × 3	0.476 ± 0.019	0.429 ± 0.020	0.428 ± 0.020	**0.452 ± 0.013**	0.520	0.388	137.1
Random CDSs (MSA)	Three frames	100000 × 3	0.409 ± 0.06	0.411 ± 0.059	0.448 ± 0.044	**0.410 ± 0.055**	0.541	0.207	108.27
	Three random CDSs	100000 × 3	0.411 ± 0.06	0.413 ± 0.059	0.447 ± 0.043	**0.412 ± 0.055**	0.540	0.201	109.47

To sidestep the effect of aligners, MSA was used to obtain the optimal alignments [38]. Unfortunately, MSA cannot be applied to align protein sequences > 500 AAs because of the memory requirements, so that it cannot be applied to many real genes. So, only the random frame/native translations were aligned using MSA. From these MSAs, the est. mean random frame similarity is 0.410 ± 0.055, but the est. mean random similarity is also as high as 0.412 ± 0.055 (Table 2 and S1a). Besides, on average, MSA placed as many as 108.3 and 109.5 gaps in the random frame and random native translations, respectively, suggesting that the false similarity estimates caused by gappy alignments cannot be avoided by using an optimal alignment algorithm.

As described in FrameAlign: aligning of frameshifts and wild-type protein sequences section, frame translations cannot be aligned correctly in a multiple sequence alignment but only in pairwise alignments. When the random frame or native translations are aligned using FrameAlign, only one gap is inserted into each frameshift translation, and no gaps are inserted into the native translations. From FrameAlign, the est. mean random similarity and est. mean random frame similarity is 0.383 ± 0.018 and 0.394 ± 0.016 (Table 3), respectively. Their difference is small but statistically extremely significant (t-test *P*-value ≈ 0). As well, the overall average of the real frame similarities is 0.450 ± 0.030 (Table 3), much higher than the est. means of random similarities or random frame similarities (t-test P-value ≈ 0), indicating that the real frame translations are more similar to each other than the random frame translations, which cannot be revealed by the similarity calculations from the ClustalW or MSA alignments. Although the confidence intervals for some of the comparisons overlap, the differences for all six comparisons are statistically extremely significant (Table S1a and S1b). Since all available coding genes were considered for each species, the standard errors are hundreds of times lower than the standard deviations due to the large sample size (number of genes).

**Table 3:** The similarities of proteins and their frameshifts (aligned by FrameAlign)

Type	Species	Number of CDSs	Average Similarity						Number of Gaps
			δ_**12**_	δ_**13**_	δ_**23**_	δ	MAX	MIN	
Real CDSs (FrameAlign)	*H. sapiens*	71,853	0.492 ± 0.043	0.472 ± 0.044	0.434 ± 0.040	**0.466 ± 0.029**	0.713	0.194	2
	*P. troglodytes*	15,781	0.491 ± 0.046	0.468 ± 0.046	0.431 ± 0.042	**0.463 ± 0.030**	0.625	0.311	2
	*M. musculus*	27,208	0.484 ± 0.046	0.469 ± 0.042	0.426 ± 0.040	**0.460 ± 0.029**	0.739	0.286	2
	*X. tropicalis*	7706	0.481 ± 0.042	0.481 ± 0.041	0.439 ± 0.037	**0.467 ± 0.028**	0.644	0.353	2
	*D. rerio*	14,151	0.471 ± 0.044	0.468 ± 0.040	0.408 ± 0.040	**0.449 ± 0.030**	0.614	0.314	2
	*D. melanogaster*	23,936	0.475 ± 0.046	0.457 ± 0.044	0.362 ± 0.047	**0.431 ± 0.030**	0.689	0.236	2
	*C. elegans*	29,227	0.450 ± 0.047	0.475 ± 0.045	0.421 ± 0.043	**0.449 ± 0.032**	0.634	0.224	2
	*A. thaliana*	35,378	0.442 ± 0.045	0.477 ± 0.044	0.412 ± 0.041	**0.444 ± 0.031**	0.882	0.244	2
	*S. cerevisiae*	5889	0.461 ± 0.041	0.510 ± 0.042	0.423 ± 0.038	**0.465 ± 0.029**	0.692	0.259	2
	*E.coli*	4140	0.435 ± 0.046	0.426 ± 0.047	0.372 ± 0.043	**0.411 ± 0.030**	0.571	0.237	2
	Average	235,269	0.468 ± 0.045	0.470 ± 0.043	0.413 ± 0.041	**0.450 ± 0.030**	0.882^a^	0.194^a^	2
Random CDSs (FrameAlign)	Three frames	100,000	0.394 ± 0.028	0.394 ± 0.028	0.395 ± 0.028	**0.394 ± 0.016**	0.477	0.330	2
	Three random CDSs	100000 × 3	0.383 ± 0.028	0.383 ± 0.028	0.383 ± 0.028	**0.383 ± 0.018**	0.458	0.304	0

^a^Very large/small similarity values were observed in a few very short or repetitive peptides

As described in Aligning and computing the similarities of wild types and frameshifts section, the average frame similarities are defined as the shiftability of protein-coding genes. As the frame similarities calculated from the ClustalW and MSA alignments are false, the mean frame similarity from FrameAlign is considered the true shiftability of coding genes. As shown in Table 3, the overall average shiftability is close to 0.45 for the real genes but less than 0.4 for the random CDSs. In other words, on average, about 45% of the amino acids remain conserved in the real frameshift translations. In addition, the shiftability varies substantially in different species, ranging from 0.411 (*E. coli*) to 0.466 (human), but the standard deviations are generally as low as 0.030 in all tested species, i.e., the shiftability is species-dependent and is concentrated at a particular value for most genes in a specific species.

For example, the readthrough frameshift translations of zebrafish *vegfaa* look different from the wild type, but ClustalW aligns them well (Fig. 1B), the est. frame similarities are 0.5233, 0.4922, and 0.4819, and the average is 0.4991 (Table S1c); when these translations are aligned by FrameAlign (Fig. 1C-F), the est. frame similarities are 0.5238, 0.4921, and 0.4043, and the average is 0.4734 (Table S1c). At first glance, these similarities seem surprisingly high, so we must emphasize that this case was not cherry-picked but arbitrarily selected for visualization. Furthermore, the frame similarities for all zebrafish coding genes average 0.4491, ranging from 0.3145 to 0.6141 (Table 3 and S1b). In zebrafish, 1520 (10.74%) of the total 14,151 coding genes have an even higher shiftability than *vegfaa*. As shown in Table S1b, high frame similarities are not rare but pretty common either in zebrafish or any other species tested. The process for computing the frame similarities is demonstrated in Table S1c. One can easily reproduce similar results with many other real coding genes.

### The genetic code was optimized for frameshift tolerance

As described in Computational analysis of frameshift codon substitutions section, the average amino acid substitution scores for random, interchangeable, and frameshift substitutions were computed. As shown in Table 4 and S2, of the 4096 possible random substitutions, only a small proportion (230/4096 = 5.6%) are synonymous, and the proportion of positive substitutions (with a positive substitution score) is 859/4096 = 20.1%. Interchangeable substitutions have the highest mean score because most (192/256 = 75%) interchangeable substitutions are synonymous, and at the same time, most (192/230 = 83%) synonymous substitutions are interchangeable. In contrast, only a small percentage (28/512 = 5.5%) of the frameshift substitutions are synonymous (Table 4), while the remaining 94.5% are nonsynonymous. But 29.7% of frameshift substitutions are positive nonsynonymous, about 1.5-fold of that in random (20.1%) and about 2-fold of that in interchangeable (15.6%). In summary, interchangeable substitutions are assigned mostly with synonymous AAs in the SGC, while frameshift substitutions are more frequently with positive nonsynonymous ones.

**Table 4:** The amino acid substitution scores for different kinds of codon substitutions

Codon Substitution	Random	Frameshift		Interchangeable
		***FF***	***RF***	
Type of Codon Substitution				
All	4096	256	256	256
Unchanged (%)	64 (1.6%)	4 (1.6%)	4 (1.6%)	64 (25%)
Changed (%)	4032 (98.4%)	252 (98.4%)	252 (98.4%)	192 (75%)
SS (%)	230 (5.6%)	14 (5.5%)	14 (5.5%)	192 (75%)
NSS-Positive (%)	859 (20.1%)	76 (29.7%)	76 (29.7%)	40 (15.6%)
NSS-Negative (%)	3007 (73.4%)	166 (64.8%)	166 (64.8%)	24 (9.4%)
Average Substitution Score				
BLOSSUM62	−1.29	−0.61	−0.65	3.77
PAM250	−4.26	−0.84	−0.84	3.68
GON250	−10.81	−1.78	−1.78	35.60

*SS/NSS* Synonymous/nonsynonymous substitution, *FF/RF* Forward/reverse frameshift substitutions

Besides, no matter which AA substitution scoring matrix is used, the average FSSs are always significantly higher than random substitutions. Using GON250, e.g., the average FSS (− 1.78) is significantly higher than the average RSS (− 10.81). As shown in Table S2, AAs assigned to frameshift substitutions are significantly more conservative than those to random substitutions. The *P*-values of the t-tests between the FSSs and the RSSs are 2.497 × 10^− 10^ (forward frameshifting vs. random substitutions) and 2.896 × 10^− 9^ (reverse frameshifting vs. random substitutions), respectively.

In the most common scoring matrices, such as BLOSSUM62, PAM250, and GON250, most scores are negative, and the percentage of positive scores is about 35%, i.e., in random codon substitutions, the percent of positive substitutions is about 35%, which is consistent with the observed mean random similarity, 0.383 (Table 3). However, as mentioned above, the mean frame similarity of the real genes is significantly higher than the means of the random similarities or random frame similarities, implying that the shiftability of genes is determined at two different levels — the genetic code and the coding sequences.

### The natural genetic code ranks at the top of all possible codon tables

**Fig. 2:**
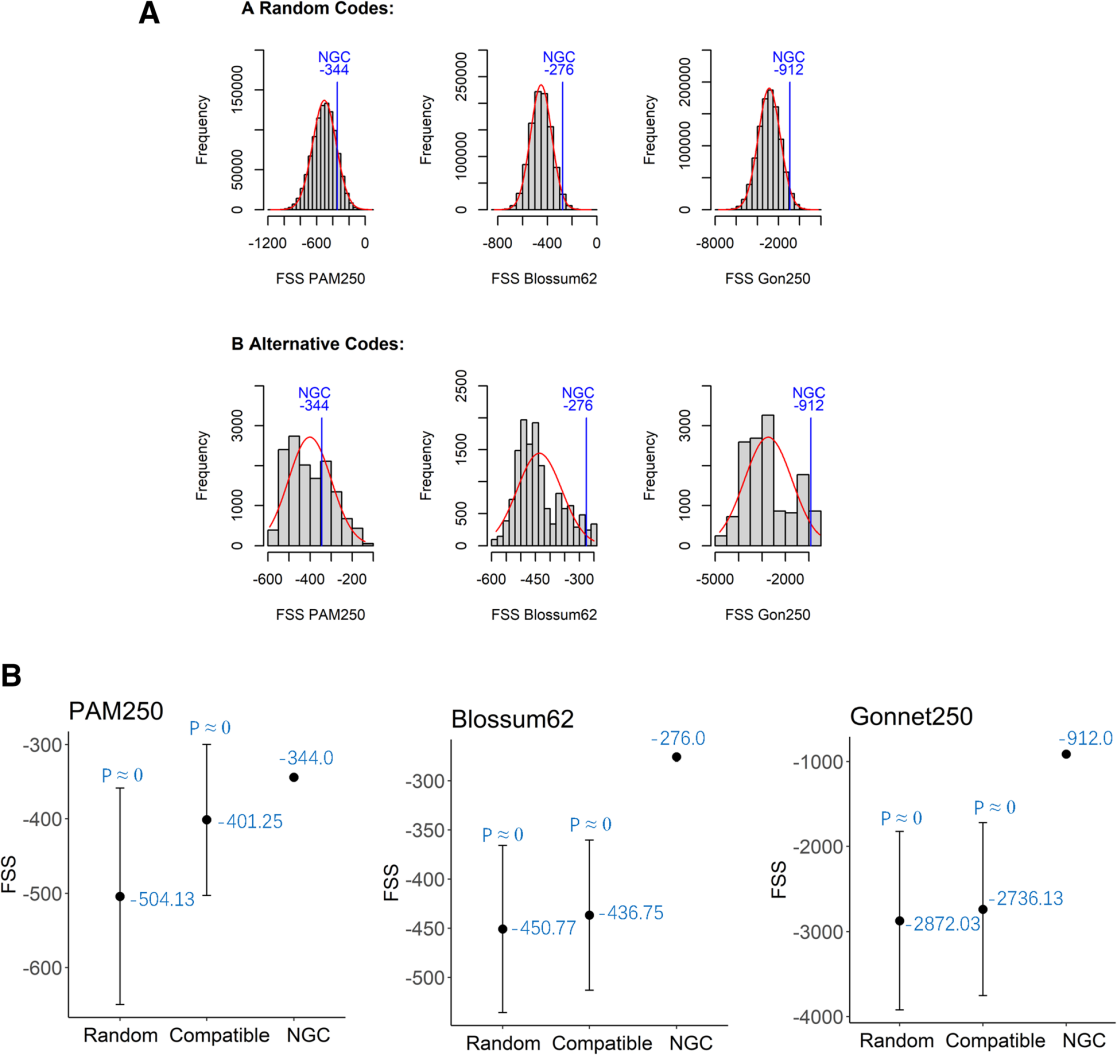
The distribution and the statistical analysis of the FSSs for the alternative genetic codes. **A** The frequencies of occurrence of the FSSs in the random codon tables and the compatible codon tables. **B** The means and standard deviations of the sum FSSs of different types of genetic codes. T-tests indicate that the sum FSS of the NGC is significantly higher than the mean FSS of the random or compatible genetic codes in all six comparisons (P ≈ 0) (Table S3). NGC: natural genetic code; FSSs were calculated using matrices PAM250, BLOSSUM62, and GON250. The probability densities were computed using a normal distribution function, and the diagrams were plotted in the language R

To further investigate the frameshift optimality of the SGC, we compared it with two types of alternative codon tables:

1. Random codon tables are produced by swapping the amino acids assigned to sense codons while keeping all degenerative codons synonymous [7]. From all possible (20! = 2.43290201 × 10^18^) random codon tables, 100 independent samples, each with 1 million codon tables, were sampled using a simple random sampling algorithm. As shown in Fig. 2A and Table 5, when FSSs were calculated using PAM250, BLOSSUM62, and GON250 scoring matrices, the sum FSS of the SGC ranks among the top 13.26, 1.98, and 2.94% in the samples, respectively. For all the 100 independent samples, the standard deviations of the means and the ranks of FSSs are as low as 0.03-0.15%, indicating that the sample size (1 million) is sufficiently large.
2. Compatible codon tables are produced by permuting the bases in the three different codon positions independently and preserving the AA assignment [8]. There are 4! (= 24) possible permutations of the four nucleotides for each codon position. All 24^3^ (= 13,824) compatible codon tables were produced, and their FSSs were computed (Table S3). Figure 2A and Table 5 show that the SGC ranks in the top 30.91% of the compatible genetic codes when their FSSs were computed using the PAM250 scoring matrix but ranks in the top 3.48% when using BLOSSUM62 or GON250.

**Table 5:** The frameshift substitution scores of the natural and alternative genetic codes

Genetic codes (Number tested)	Scoring Matrix	FSS of the natural genetic code (NGC)					FSS of the alternative genetic codes		
		FSS Score	Rank	Rank%	STDEV	STDEV%	Average	STDEV	STDEV%
Random (1,000,000 × 100)	PAM250	− 344	132,586.79	13.26%	1011.17	0.1011%	− 504.88	0.54	−0.1073%
	Blossum62	− 276	19,752.52	1.98%	295.17	0.0295%	−450.53	0.27	−0.0598%
	Gonnet250	−912	29,447.26	2.94%	398.72	0.0399%	− 2872.95	4.16	−0.1447%
Compatible (13824)	PAM250	−344	4273	30.91%	–	–	−401.25	–	–
	Blossum62	−276	481	3.48%	–	–	−436.75	–	–
	Gonnet250	−912	481	3.48%	–	–	− 2736.13	–	–

In either case, the ranks of the SGC computed using BLOSSUM62 and GON250 are highly consistent with each other, indicating that the SGC ranks in the top 2.0–3.5% of all possible codon tables in terms of frameshift tolerability. Moreover, the t-tests *p*-values are close to zero in all six comparisons (Table S3), suggesting that the sum FSS of the SGC is significantly higher than the mean FSS of the random or compatible genetic codes (Fig. 2B). Itzkovitz and Alon [8] pointed out that, due to the wobble constraint for base pairing in the third position, only two permutations (the identity permutation and the A↔G permutation) are allowed in the third position. Thus, the genetic code has only 24 × 24 × 2 = 1152 distinct alternatives. Of these unique codes, only a dozen to a few dozen are superior to the natural genetic code regarding frameshift tolerance. Therefore, it is concluded that the SGC is nearly optimal in terms of frameshift tolerance.

### The shiftability was further optimized at gene−/genome-level

As abovementioned, shiftability is species-dependent (Table 3). For some real genes, shiftability is exceptionally high (Table S1b), such as *E. coli ydaE* (*δ =* 0.571) and the human glutenin gene (*δ =* 0.660). As shown in Table 6 and S4, the mean FSS weighted by codon usages in *E. coli*, *A. thaliana*, and *C. elegans* are lower than expected (the mean FSSs of the equal usage of codons), showing that frameshift-resistant codons (FRCs) are not overrepresented in these genomes. The weighted mean FSSs are significantly higher than expected in humans, mice, *Xenopus*, and yeast, suggesting that FRCs are overrepresented in these genomes. In other words, the shiftability of certain genes or genomes can be adjusted through the biased usage of codons.

**Table 6:** The usage of codons and their weighted mean FSSs (Gon250)

No	Species (Codon Usage)	Weighted mean FSS
1	*H. sapiens*	−9.82
2	*M. musculus*	−13.47
3	*X. tropicalis*	−12.75
4	*D. rerio*	−20.58
5	*D. melanogaster*	−19.43
6	*C. elegans*	−23.38
7	*A. thaliana*	−22.52
8	*S. cerevisiae*	−14.08
9	*E. coli*	−28.59
10	Equal usage	−22.27

On the other hand, frameshifting involves adjacent codon pairs, so the usages of codon pairs are more likely to be related to the frameshift tolerance of genes. As shown in Table 7 and S5, the usages of codon pairs are also highly biased in all species tested. Surprisingly, of the 4096 codon pairs, less than 41% (up to 1660) are overrepresented, while the remaining 59 + % (> 2400) codon pairs are underrepresented or even unused, suggesting that the synonymous codon pairs had undergone a strong selection pressure [39]. The weighted mean FSSs are significantly lower than expected (the mean FSS of equal usage of codon pairs) in *E. coli, C. elegans,* and *A. thaliana*, showing that frameshift-resistant codon pairs (FRCPs) are not overrepresented in these genomes; in humans, mice, *Xenopus*, and yeast, however, the weighted mean FSSs are significantly higher than expected, indicating thatFRCPs are overrepresented in these species. In these higher species, genome-level shiftability is also higher than those in the lower species (Table 3), suggesting that the shiftability is related to the usage of codons and codon pairs.

**Table 7:** The usage of codon pairs and their weighted mean FSSs (Gon250)

No	species	Number of codon pairs			Weighted mean FSS		
		Over-represented	Under-represented	Absent	Over-represented	Under-represented	All
1	*H. sapiens*	1573	2523	50	−1.52	−7.80	−3.06
2	*M. musculus*	1505	2591	190	−2.83	−7.13	− 3.81
3	*X. tropicalis*	1660	2436	148	−3.12	−6.98	−3.80
4	*D. rerio*	1493	2603	148	−4.87	−6.09	−5.18
5	*D. melanogaster*	1418	2678	140	−5.33	−5.86	−5.02
6	*C. elegans*	1469	2627	164	−6.47	−5.26	−6.11
7	*A. thaliana*	1566	2530	15	−6.30	−5.35	− 6.37
8	*S. cerevisiae*	1493	2603	159	−4.86	−6.14	−4.27
9	*E. coli*	1389	2707	197	−6.76	− 5.11	− 6.82
10	Equal Usage	0	0	0	N/A	N/A	−5.67

## Discussion

### The optimality of the genetic code and the shiftability of coding genes

Since the origin of life, the natural genetic code has existed and has been optimized by codon reassignments and competition with alternative codes [40]. The natural genetic code was optimized along with several properties during the early history of evolution [41]. It has been reported that the natural genetic code was optimized for the minimization of translational errors, which is explained by the selection to minimize the deleterious effects of translation errors [4]. Besides, it was suggested that only one in every million alternative genetic codes is more efficient than the SGC in minimizing the effects of point-mutations or translational errors [7]; Also, it was shown that the genetic code is nearly optimal for storing additional information within coding sequences, such as out-of-frame hidden stop codons (HSCs) [8].

During 2000-2014, only a few reports were published on the optimality of the genetic code [42–45]. Since we proposed the hypothesis that the natural genetic code was optimized for frameshift tolerance in 2015 [25], this topic has gained renewed attention, with over a dozen new reports emerging on the optimality of the genetic code [26, 46–57]. These results are generally more reliable, informative, and supportive of the early conclusions that the SGC was optimized regarding the robustness to the effects of point mutations or frameshift mutations. Particularly, using evolutionary algorithms, Wnętrzak, Błażej, and Mackiewicz proved that the SGC was optimized in both point and frameshift mutations [50–52].

A complete frameshift is usually a loss of function, while a functional frameshift is usually a partial frameshift. Shiftability does not guarantee that all frameshifts retain their wild-type function but have a higher probability of restoring normal structure and function when repairing a frameshift mutation [58]. Because of the shiftability, on average, near half of the amino acids remain conserved in a frameshift, regardless of whether it is a complete or a partial frameshift and where the frameshifting starts and ends. It is conceivable that a genetic code with a greater shiftability had a better chance of winning the competition with its competitors in earlier evolutionary history. As mentioned above, on average, about 40 to 45% of the amino acids are kept conservative in a frameshift. This intriguing feature of the genetic code forms the basis of frameshift tolerance, which explains why functional frameshifts exist [17, 21, 59].

Moreover, if a frameshift is not removed by selecting against, it can be repaired by a reverse mutation or changed by point mutations [60]. Proteins have been evolving through point and frameshift mutations in their CDSs. The point mutation rate is extremely low, so that they alter the sequences, structures, and functions of proteins at a slow rate. However, frameshift + point mutations provide a far more effective means for fast-evolving protein sequences, allowing the emergence of novel (or overlapping) genes or protein domains. Undoubtedly, shiftability can play a vital role in the evolutionary process in maintaining, repairing, and evolving proteins and their coding genes.

With billions of years of evolution, the canonical genetic code remains a fundamental outline that is highly conserved across all three domains of life [61]. On the other hand, the natural genetic code results from the coevolution along with the ribosome complex [62]. It has been confirmed that codon reassignments to amino acids exist in the alternative genetic codes [63–65], suggesting that the genetic codes have undergone many rounds of optimization in the evolution history. The alternative codes are slightly different from the SGC; however, it remains to be clarified whether these minor changes significantly affect the shiftability of their coding genes and genomes.

### The usage of codons and codon pairs

There have been quite some disputes on the cause and consequence of the usages of codons/codon pairs, such as gene expression level [66], mRNA structure [67], mRNA stability [68], and protein abundance [69]. Here we demonstrated that the shiftability of a gene or a genome is adjusted through the usage of codons and codon pairs, e.g., the overall average shiftability for all protein-coding genes is significantly higher in humans (0.4660) than in fruit flies (0.4311) (Table 3). Meanwhile, the weighted average FSSs is also significantly higher in humans (− 3.06) than in fruit flies (− 5.02) (Table 7). Together, these data suggest that many genes in the human genome were optimized for frameshift tolerance and that the shiftability of coding genes could either be a cause or a consequence of a biased usage of codons or codon pairs. The more a frameshift resembles the wild type, the more likely it can restore a normal function when it encounters a frameshift mutation. Thus, overuse of frameshift-resistant codons or codon pairs confers an evolutionary or survival advantage on a gene or genome. In other words, frameshift tolerance is achieved not only through the optimality of the genetic code but, more importantly, by further optimizing genes and genomes through biased usages of codons/codon pairs, which sheds light on the role of frameshift mutations in molecular and genomic evolution.

### The statistics for measuring frameshift tolerability

We calculated frameshift substitution scores and showed that they are significantly higher than random substitution scores. Recently*,* Bartonek, Braun, and Zagrovic analyzed frameshift proteins using the amino acids’ physicochemical properties (PCPs) [26]. From a chemical point of view, PCPs are more suitable for analyzing frameshift tolerance with consideration of protein structures, while FSSs are more convenient for biological studies. Substitution scores are calculated from the probability that different amino acids were substituted by each other over time. Although the substitution scores are ultimately determined by the physicochemical properties of the amino acids, their values also reflect the evolutionary relationships among the organisms of interest. As such, they are widely used in sequence analyses, such as calculating similarities, constructing alignments, and searching databases. Each family of scoring matrices has different members, such as PAM1, …, PAM100, and PAM250, representing substitution probabilities over different timescales. Different scoring matrix members are designed for different evolutionary distances, e.g., PAM1, …, PAM100 are more suitable for aligning closely related protein sequences, while PAM250 is more suitable for remotely related sequences. Pearson [70] pointed out that “deep” scoring matrices (like BLOSUM62) target alignments with 20-30% identity, while “shallow” scoring matrices (e.g., VTML10), target alignments that share 50-90% identity, reflecting much less evolutionary change. The alignment of frameshifts is unique and special because a frameshift and its wild-type CDS are closely related, but their translations have a low identity and a moderate similarity. Obviously, “deep” matrices are more suitable than “shallow” matrices for aligning and analyzing frameshifts. This study adopted three representative “deep” matrices to calculate FSSs. Since frame similarities are quasi-constant, these scoring matrices were used without considering divergence levels. However, it remains undetermined which scoring matrix family (or a family member) is best suited for calculating frameshift tolerance, or whether a specialized scoring matrix is needed to analyze frameshift mutations.

### The readthrough rules and their impact on the computation of similarity

This study incorporates computational frameshifting and readthrough into the analysis. It is important to note that such computational operations are conceptually different from biological frameshifting and translational readthrough. They do not require that they truly occur in an organism because these operations are used only for calculating similarities. So, in the present study, they are not taken as biological laws but computational methods borrowed from biology. However, the expected proportion of hidden stop codons (HSCs) in the frameshifted CDS s is 3/64 = 4.69%, and the proportion of HSCs in real genes may even be higher than expected [9]. Therefore, the readthrough rules can significantly affect the frame similarity calculations. We have conducted a series of data analyses and found that the location and distribution of HSCs and the matching wild-type amino acids in real genes are not random, different from the simulated random CDSs.

Therefore, the differences between readthrough and non-readthrough translations are not negligible. All these data suggest that the readthrough rules are probably adapted to the genetic code and explain part of its optimality. As the presentation of these results depends on the present study, we will present these data in another article.

## Conclusion

Based on the above analysis, we conclude that the genetic code, many genes and certain genomes were optimized for frameshift tolerance. Shiftability ensures high similarities between frameshifts and their wild-type counterparts, endowing coding genes the inherent tolerability to frameshift mutations in either forward or reverse direction. Thanks to this unique property, the natural genetic code obtained excellent fitness better than its competitors, thus winning the competition in the early evolution. The shiftability serves as an innate mechanism by which coding genes and genomes tolerate frameshift mutations, and thus, deleterious frameshift mutations could have been utilized as a driving force for molecular evolution. However, the impacts of frameshift tolerance on molecular or genomic evolution remain to be characterized across the tree of life.

## Supplementary Information

**Additional file 1: Table S1a.** Frame similarities aligned by ClustalW or MSA. (1) The summary of the similarities of natural or simulated proteins and their frameshifts aligned by ClustalW or MSA (Table 2). (2) The similarities of random frame translations (aligned by ClustalW). (3) The similarities of random frame translations (aligned by MSA). (4) The similarities of *E. coli* frame translations (aligned by ClustalW). (5) The similarities of yeast frame translations (aligned by ClustalW). (6) The similarities of human frame translations (aligned by ClustalW). (7) The similarities of chimpanzee frame translations (aligned by ClustalW). (8) The similarities of mouse frame translations (aligned by ClustalW). (9) The similarities of Xenopus frame translations (aligned by ClustalW). (10) The similarities of zebrafish frame translations (aligned by ClustalW). (11) The similarities of fruit fly frame translations (aligned by ClustalW). (12) The similarities of nematode frame translations (aligned by ClustalW). (13) The similarities of Arabidopsis frame translations (aligned by ClustalW). **Table S1b.** Frame similarities aligned by FrameAlign. (1) The summary of the similarities of natural or simulated proteins and their frameshifts aligned by FrameAlign (Table 3). (2) The similarities of random translations (aligned by FrameAlign). (3) The similarities of *E. coli* frame translations (aligned by FrameAlign). (4) The similarities of yeast frame translations (aligned by FrameAlign). (5) The similarities of human frame translations (aligned by FrameAlign). (6) The similarities of chimpanzee frame translations (aligned by FrameAlign). (7) The similarities of mouse frame translations (aligned by FrameAlign). (8) The similarities of Xenopus frame translations (aligned by FrameAlign). (9) The similarities of zebrafish frame translations (aligned by FrameAlign). (10) The similarities of fruit fly frame translations (aligned by FrameAlign). (11) The similarities of nematode frame translations (aligned by FrameAlign). (12) The similarities of Arabidopsis frame translations (aligned by FrameAlign). **Table S1c.** Computing frame similarities of zebrafish vegfaa. (1) The three frame translations are aligned by ClustalW. (2) The three frame translations are aligned by FrameAlign. (3) The Gon250 scoring matrix used in (1) and (2).

**Additional file 2: Table S2.** FSSs of the natural genetic code. (1) The FSSs of the natural genetic code using Scoring Matrix Gon250. (2) The FSSs of the natural genetic code using Scoring Matrix Blossum62. (3) The FSSs of the natural genetic code using Scoring Matrix PAM250.

**Additional file 3: Table S3.** FSSs of the alternative genetic codes. (1) The summary of the FSSs of the natural and alternative genetic codes (Table 5). (2) Comparing the FSSs of the standard genetic code to random or compatible alternative genetic codes. (3) The FSSs of the random genetic codes. (4) The FSSs of the compatible genetic codes (PAM250). (5) The FSSs of the compatible genetic codes (Blossum62). (6) The FSSs of the compatible genetic codes (GON250).

**Additional file 4: Table S4.** FSSs of different codon usages. (1) The summary of codon usages and their weighted mean FSSs (Table 6). (2) The codon usages and their weighted mean FSSs of humans. (3) The codon usages and their weighted mean FSSs of mouse. (4) The codon usages and their weighted mean FSSs of xenopus. (5) The codon usages and their weighted mean FSSs of zebrafish. (6) The codon usages and their weighted mean FSSs of fruit fly. (7) The codon usages and their weighted mean FSSs of nematode. (8) The codon usages and their weighted mean FSSs of Arabidopsis. (9) The codon usages and their weighted mean FSSs of yeast. (10) The codon usages and their weighted mean FSSs of *E. coli*. (11) The 64 triplet codons and their FSSs.

**Additional file 5: Table S5.** FSSs of different usages of codon pairs. (1) The summary of codon pair usages and their weighted mean FSSs (Table 7). (2) The codon pair usages and their weighted mean FSSs of humans. (3) The codon pair usages and their weighted mean FSSs of mouse. (4) The codon pair usages and their weighted mean FSSs of xenopus. (5) The codon pair usages and their weighted mean FSSs of zebrafish. (6) The codon pair usages and their weighted mean FSSs of fruit fly. (7) The codon pair usages and their weighted mean FSSs of nematode. (8) The codon pair usages and their weighted mean FSSs of Arabidopsis. (9) The codon pair usages and their weighted mean FSSs of yeast. (10) The codon pair usages and their weighted mean FSSs of *E. coli*. (11) The equal codon pair usages and their weighted mean FSSs.

## Data Availability

The datasets analyzed during the current study are available in the GenBank Genome Database, https://www.ncbi.nlm.nih.gov/genome/, including the reference genome sequences of *Escherichia coli* str. K-12 substr. MG1655 (NC_000913.3), *Saccharomyces cerevisiae* S288C (assembly R64)*, Caenorhabditis elegans* (assembly WBcel235)*, Arabidopsis thaliana* (assembly TAIR10.1)*, Drosophila melanogaster* (assembly Release 6 plus ISO1 MT)*, Danio rerio* (assembly GRCz11)*, Xenopus tropicalis* (assembly UCB_Xtro_10.0)*, Mus musculus* (assembly GRCm39), *Pan troglodytes* (assembly Clint_PTRv2), and *Homo sapiens* (assembly GRCh38.p13). The Supplementary Information and Supplementary Tables are available online along with this article at the website of BMC genomics. The source code of the java programs used to analyze the data are available at GitHub (https://github.com/CAUSA/Frameshift).
